# 

**Published:** 1831-12

**Authors:** 


### MONTHLY LIST OF MEDICAL BOOKS.

[*Medical Works cannot be entered on this List unless a copy be sent for the purpose, the titles of Books having frequently been sent to us as published which have not been published for weeks, or even months, after.*]

A Critical and Experimental Essay on the Circulation of the Blood; especially as observed in the Minute and Capillary Vessels of the Batrachia and of Fishes. By Marshall Hall, M.D., F.R.S. E., M.R.I., M.Z.S., &c.—8vo. pp. 187; plates. Seeley and Burnside, London.

A Practical Medico-Historical Account of the Western Coast of Africa: embracing a Topographical Description of its Shores, Rivers, and Settlements, with their Seasons and comparative Healthiness: together with the Causes, Symptoms, and Treatment of the Fevers of Western Africa; and a similar Account respecting the other Diseases which prevail there. By James Boyle, M.C.S. L., Colonial Surgeon to Sierra Leone, Surgeon R.N., &c.—8vo. pp. 423. Highley, London.

An actually practised and effectually successful Mode of Treatment of the Cholera. Translated from a Letter of Dr. Ewertz, Practitioner of Dünæburg, in European Russia; addressed to Baron E. F. Von Graefe, Body-Surgeon to the King of Prussia.—8vo. pp. 8. Schloss, London.

Cases of Insanity; with Medical, Moral, and Philosophical Observations and Essays upon them. By M. Allen, m.d. &c. Part I. Vol. I. This first part contains an Essay on Atmospheric, the Seasons, Diurnal, Lunar, and Planetary Influence; with whicb is incorporated an Inquiry into the Cause of Epidemics, and of Cholera Morbus in particular.—8vo. pp. 212. Swire, London.

A Practical Guide to Operations on the Teeth: to which is prefixed, an Historical Sketch of the Rise and Progress of Dental Surgery. By James Snell, Dentist, Member of the Royal College of Surgeons; Lecturer on the Anatomy and Diseases of the Teeth, &c.—8vo. pp. 207. Wilson, London.

Elements of Surgery. By Robert Liston, Fellow of the Royal Colleges of London and Edinburgh; Surgeon to the Royal Infirmary, &c. Part II.—8vo. pp. 334. Longman, London.

A Synopsis of the Bones, Ligaments, Muscles, Blood-vessels, and Nerves of the Human Body. By Wm. Sands Cox, Surgeon to the Dispensary, and Lecturer on Anatomy and Surgery at the School of Medicine in Birmingham.—8vo. pp. 310. Longman, London.

Operative Surgery. Maingault's Illustrations of the different Amputations performed on the Human Body, represented by Plates designed after Nature; with Alterations and Practical Observations. By Wm. Sands Cox, Lecturer on Anatomy and Surgery at the Birmingham School of Medicine, and Surgeon to the General Dispensary.—Folio. Longman, London.

Observations on Cholera, as it appeared at Port-Glasgow, during the Months of July and August, 1831. Illustrated by numerous Cases. By John Marshall, m.d.—8vo. pp. 80. Whittaker and Co., London.

Pharmacopœia Medico-Chirurgica in usum Medicinam Facientium, a Roberto G. Holland, Chirurgo, Concinnata, Edinburghi: veneunt apud Joannem Carfræ et Filium.—8vo. pp. 186. Londini, apud Longman.

Researches to establish the Truth of the Linnæan Doctrine of Animate Contagion: wherein the Origin, Causes, Mode of Diffusion, and Cure of Epidemic Diseases, Spasmodic Cholera, Dysentery, Plague, Smallpox, Hooping Cough, Leprosy, &c. are illustrated by Facts from the Natural History of Mankind, of Animals, and of Vegetables, and from the Phenomena of the Atmosphere. By Adam Neale, m.d., Physician to his Majesty's Forces during the Peninsular War, and one of the Physicians Extraordinary to his late Royal Highness the Duke of Kent, &c.—8vo. pp. 258. Longman, London.

The History of the Contagious Cholera; with Facts explanatory of its Origin and Laws, and of a Rational Method of Cure. By James Kennedy, Member of the Royal College of Surgeons, London.—8vo. pp. 291. Cochrane, London.

Rules and Regulations proposed by the Board of Health for the Purpose of Preventing the Introduction and Spreading of the Disease called Cholera Morbus; the early Symptoms of the Disease in its most marked Form, as it occurred to the Observation of Dr. Russel and Dr. Barry at St. Petersburg, and corroborated by the Accounts from other Places where the Cholera has prevailed; with the most approved Treatment. Published by a Committee of the Lords of his Majesty's Privy Council. To which are added, a Warning to the British Public against the alarming Approach of the Indian Cholera, by Sir Gilbert Blane, Bart. f.r.s.; and the History of the Epidemic Cholera of India, from its first Appearance in the Year 1817 to 1831, and the dreadfnl Ravages it has committed, with the Dates of its first Appearance in the different Provinces.—Burgess and Hill, London.

Elements of Botany: consisting of the Anatomy and Physiology of Plants; an Explanation of the Classification of Plants by Linnæus; an Arrangement of Medicinal Plants, and a Glossary of Botanical Terms. Second Edition; with Plates. By John Steggall, M.D. &c.—12mo. pp. 155. Highley, London.

A Memorial presented to the Medical and Surgical Officers of the Worcester, Salop, Birmingham, Gloucester, and Hereford Infirmaries, on the Abuses existing in the Public Hospitals; with an Appendix, containing the Answers received from them.—Renshaw and Rush, London.

The Principles and Practice of Obstetric Medicine, in a Series of Systematic Dissertations on Midwifery, and on the Diseases of Women and Children. Illustrated by numerous Plates. By David D. Davis, M.D. M.R.S.L., Professor of Midwifery in the University of London, &c.—4to. pp. 24. John Taylor, London.

Remarks on the Subject of Lactation: containing Observations on the Healthy and Diseased Conditions of the Breast-milk; the Disorders frequently produced in Mothers by Suckling; and numerous illustrative Cases, proving that, when protracted, it is a common Cause, in Children, of Hydrencephalus, or Water in the Brain, and other serious Complaints. By Edward Morton, M.D. Cantab. &c.—8vo. pp. 63. Longman, London.

Practical Remarks on the Disease called Cholera, which now exists on the Continent of Europe. By John Goss, Surgeon, and late Assistant Surgeon in the Hon. East India Company's Service, Bombay Establishment.—8vo. pp. 27.—Longman, London.

Remarks on the Cholera Morbus: containing a Description of the Disease, its Symptoms, and Treatment; together with Suggestions as to the best Means of guarding against its Attack: submitted to the Attention of the Profession, but designed principally for the use of the Public in general. By H. Young, M.D., formerly of the H. E. I. C. Medical Service in Bengal.—8vo. pp. 78. Smith, Elder, and Co. London.

The Polish Revolution of 1830, 1831; with Sketches of the leading Characters.—8vo. pp. 104. Cochrane and Co. London.

Observations on the Nature and Treatment of the Cholera Morbus, now prevailing epidemically in St. Petersburg. By G. W. Lefebre, M.D., Physician to the British Embassy, St. Petersburg.—8vo. pp. 96. Longman, London.

Elements of Chemistry, including the recent Discoveries and Doctrines of the Science. By Edward Turner, M.D., F.R.S. L. and E., Sec. G.S., Professor of Chemistry in the University of London, &c. Third Edition, enlarged, and carefully revised.—8vo. pp. 908. Taylor, London; Maclachlan, Edinburgh.

☞ We shall shortly give an ample Account of this excellent work.

The Elements of Chemistry, familiarly explained and practically illustrated. Part the First: Attraction, Heat, Light, Electricity.—8vo. pp. 318. John Murray, London.

An Essay on the Epidemic Cholera of India. By Reginald Orton, Surgeon H.P., late of his Majesty's 34th Regiment of Foot. Second Edition, with a Supplement and Map.—8vo. pp. 488. Burgess and Hill, London.

The London Manual of Medical Chemistry: comprising an interlinear Verbal Translation of the Pharmacopœia, with extensive Chemical, Botanical, Therapeutical, and Posological Notes, not only in reference to the Medicines enumerated in that Work, but also to those which have been recently introduced in Practice; together with the Treatment and Tests of Poisons, and an Introduction containing the Theory of Pharmaceutical Chemistry, &c. For the use of Students. By Wm. Maugham, Surgeon, and Lecturer on Chemistry and Materia Medica.—12mo. pp. 492. Whittaker, Treacher, and Co. London.

☞ The objects of this work are fully described in its ample title-page. We have looked carefully through it, and warmly recommend it as an excellent elementary guide for medical students.

A Treatise on the Diseases of the Heart and Great Vessels; comprising a new View of the Physiology of the Heart's Action, according to which the Signs, both ordinary and stethoscopic, are explained; by J. HOPE, M.D., Member of the Royal College of Physicians, &c.—8vo. pp. 600; seven diagrams. Kidd, London.

A Practical Compendium of Midwifery; being the Course of Lectures on Midwifery, and on the Diseases of Women and Infants, delivered at St. Bartholomew's Hospital, by the late ROBERT GOOCH, M.D. Prepared for publication by GEORGE SKINNER, M.R.C.S.—12mo. pp. 358. Longman, London.

### NOTICES.

*Communications have been received from* Mr. J. FOOTE, jun., Mr. LAIDLAW, *&c.*

*We have received, from our Correspondent,* Mr. M., *of L— C— street, two or three communications on the subject of Broussais and Clutterbuck's doctrines of Fever: as we are of opinion that the profession is by this time fully aware of the extent to which these doctrines may be safely applied in practice, we deem it inexpedient to publish the papers referred to. We are much indebted to* Mr. M. *for the valuable* practical *papers he has furnished us with. The MSS. remain with the Editor, should* Mr. M. *wish to have them returned.*

### INDEX

### TO THE ELEVENTH VOLUME OF THE NEW SERIES

OF

### THE LONDON MEDICAL AND PHYSICAL JOURNAL.

PAGE

ABSORBENTS of the uterus, inflammation of, in puerperal women 323
Amputation, on immediate, after injuries . . 223
Amputations, notice of "Illustrations of different" . 434
Aneurism, femoral; ligature of the artery . . 39
———, cases of . . 121
———, cases of femoral, opened by mistake . . 352
———, case resembling, from exostosis of the first rib . 40
———, dissection of a patient three years after the external iliac had been tied . 291
Angina pectoris, pathology of . 30
Apothecaries' Hall, regulations of 530
Arsenic, poisoning from topical use of . . . 265
Arteries, on the action of the . 341
Auscultation the only unequivocal evidence of pregnancy . 233
———, notice of Dr. Spittal's treatise on . . . 433

Bees, on the habits of the Mexican domestic . . 82
Blood, on the nature and cause of the buffy coat of . . 188
Botany, Dr. Steggall's Elements of, 522
Brain, cases shewing the difference between concussion and compression of . . 221
——, loss of, with fracture of cranium: recovery . . 261
——, case of fungus hæmatodes of 400
——, compression of, without stertorous breathing . 219
Brookes, Joshua, F.R.S. &c., dinner in honour of . . 89
Bursa, diseases of, of the patella 41

Cancer, treatment of, by compression . . 269

PAGE

Canine Madness, analysis of Mr. Youatt's essay on . 144
———, symptoms of . 145
Celsus, notice of Mr. Lee's edition of . . . 433
Cerebellum, child deprived of, and still retaining all its faculties 248
Cerebral irritation producing hysteria . . . 77
Chemistry, notice of a work on the Elements of . . 524
Chlorate of lime useful in purulent ophthalmia . . 251
———, a preservative against measles and smallpox 253
Cholera, report of the physicians 84
———, experiment proposed to determine whether it can be imported by merchandize . 248
———, opinions of the ancients concerning . . 363
———, precautionary methods, &c. of the "Central Board of Health" . . 527
———, proofs of the contagious nature of . . 448, 532
———, common salt given with success in Russian . 267
———, Indian, calomel useful in 285
———, bleeding to be cautiously practised in . 284
———, cases of Indian . 275
———, Dr. Barry's account of the Russian . . 267
———, vomiting and purging the least important symptoms in the Russian . . 267
———, cases of, at Glasgow . 431
———, Mr. Searle's treatise on 46
———, treatment of acute . 509
———, danger of *moist* heat in certain cases of . 511
———, progress of, exterior to Hindostan . . 511
———, laws of . 512

PAGE
Cholera of Russia, analysis of a work on . . 242
———, Indian, preservative regimen against attacks of . ib.
———, fumigations incapable of preventing the spread of . 244
———, not influenced by weather ib.
———, identity of Russian and Indian . . ib.
———, secretion and excretion of bile suspended in . 245
——— chiefly affects the poor ib.
———, probable derivation of the term . . ib.
——— contagious? . 246
———, review of works on . 504
———, variety of names applied to Indian . . 505
———, division of stages of . 506
———, advantage of bleeding in ib.
———, non-contagious form of, known for ages in Bengal . 508
———, contagious, began at Jessore in 1817 . ib.
———, differs in its severity in different individuals and different localities . . 509
———, principal arguments of the non-contagionists answered . 534
———, preliminary steps advised to be taken on the first appearance of, by the Board of Health 355
———, description of Russian 356
———, despatches relative to Russian . 182, 183
Climacteric disease, Sir H. Halford on . . . 130
Colchicum in rheumatism . 172
College of Surgeons, prizes offered by . . 265
Confectionary, on poisoned . 178
Congestion distinguished from inflammation . . 346
Constipation for nearly nine weeks 401
Cornea, nerves of, discovered . 247
Coryza phlegmatica, remarkable case of . . 249
Cowpox and smallpox, identity of, proved by curious experiments 525
Cranium, fracture of, with loss of brain: recovery . 261

Diarrhœa relieved by sulphate of copper and opium . . 108
Dyspepsia, on the sympathetic disorders and consecutive diseases of . . 478
————, on the influence of temperament in modifying . 69

PAGE
Education, Mr. Guthrie on medical . . 444
Electricity, influence of atmospheric, on the eyes . . 76
Emphysema from different causes 292
Ergot of rye, use of, has not increased the number of stillborn children . . 258
Erysipelas, remarks on the history of . . . 116
Eye, wound of, and portion of iris lost; vision not being impaired 457
Eyes, influence upon, from atmospherical electricity . 76
——, purulent inflammation of, relieved by chlorate of lime . 251

Fever, account of a contagious, generated from local causes . 331
—— cured by powder of holly-leaves . . 252
Fœtus, life of, in utero, detected by auscultation . . 234
Fractures, proposed to be treated by applying a case of plaster of Paris . . 359, 448
Fracture box, description of a new 91
French and English practice, comparison between . 302

Gibraltar epidemic, remarks on 1, 96
Glottis, on spasm of, from various causes . . . 421
Gonorrhœa, chlorate of lime recommended in . 170
————, use of Arg. Nit. Ung. in . . . 259
Goose grease, poisoning by . 76

Head, cases of injuries of . 219
——, notice of a Practical Treatise on Injuries of . 522
Heart, case of "probable" dislocation of . . 440
———, review of Dr. Hope's work on Diseases of . 513
———, diseases of, frequently curable in their early stages . 515
———, precise position of, in relation to surrounding parts . ib.
———, sounds heard during systole and diastole of ventricles of, ascribed to motions of contained fluid . . 518
———, valvular disease of . 519
———, sounds or murmurs produced in, without organic disease 520
———, on the action of the . 341
Hemorrhagic tendency in a family 437
Hernia, Mr. Mayo's clinical lecture on . . 485

PAGE
Hernia, strangulated, reduced by application of belladonna . 264
———, case of, with peritonitis, &c. 407
——— strangulated, from an injury by the patient's watch 408
———, strangulated scrotal, with encysted hydrocele leading to an incorrect diagnosis . 314
Hæmatemesis from disease of liver 349
Hooping cough, pathology and treatment of . . 254
Hydrocele, case of encysted, taken for hernia . . 315
Hydrophobia, a symptom of rabies in the dog, not in man . 147
Hydrothorax in an infant . 163
Hysteria dependent upon cerebral and spinal irritation . 77
Hysteria with bloody sweat . 249

Inanition, death from . 436
Inflammation and congestion distinguished . . 346
———, action of capillaries weakened in . . 344
Intestinal irritation likely to be mistaken for peritonitis . 320
Iris, portion of, lost from wound; vision unimpaired . 457
Iron, mode of distinguishing between the meconate and sulphocyanate of . . 179
Issues, &c., discharge from, maintained by an ointment of mezereon root . . 264
Italian medical institutions and practice . 301, 381, 461

Joints, on excision of diseased . 60

King's College, entrance of students to . . 266

Lithotomy, case of . 41
Lithotrity, advantages of . 411
———, Baron Heurteloup's improvements in . 413
——— not so applicable to children as adults . . 415
———, state of patient to be considered in the operation of ib.
———, principles of . 410
———, conflicting statements respecting . . 258
Liver, disease of, producing hæmatemesis . . 349

Measles, chlorate of lime a preservative against . 253
Medical Jurisprudence, review of Dr. Ryan's Manual of . 492

PAGE
Medicine, history of . 89
———, on the first principles of 337
———, on the principles of . 203
Melanose collections, chemical analysis of . . 226
———, mode of formation of, unknown . 227
Melanosis, disease of lungs resembling . . 402
———, case of . . 224
——— formerly confounded with other diseases . 226
Mercury, *modus curandi* of . 348
Mercurial vapours, effects of, on certain artisans . 394
Mezereon ointment, to maintain discharge from issues, &c. . 264
Midwifery cases, report of . 108
Milk, infection from, of a woman suffering from fever . 249

Narcotism from absorption . 435
Nash, Mr. D. W.; part of his examination at the London University . . 86
Nerve, section of sciatic, successful in case of neuralgia . 263
Nervous tissue, on regeneration of 435

Œdema from obliteration of veins 109
Œsophagus, case of foreign body in 451
Ophthalmia, utility of chlorate of lime in purulent . . 251
———, case of gonorrhœal 489
———, conjunctival, use of Ung. Arg. Nit. in . 259
Ophthalmic Hospital, meeting of governors of the Royal Westminster . . 180
Opium, external use of, in inflammatory diseases . 192
———, on the effects of, on inflammation, externally applied 23
Ovaria, inflammation of, in puerperal women . . 320

Patella, diseases of the bursa of 41
Peritonitis occasionally confounded with intestinal irritation . 320
———, fatal case of subacute, without well marked symptoms 42
Pertussis, pathology and treatment of . . . 254
Pharynx, case of needle lodged in 453
Phlebitis, fatality of uterine . 168
Physiology, Outlines of (review) 227
Plants, Withering's arrangement of British . . 429
Poisoned confectionary . 178
Poisoning from topical use of arsenic . . 265

PAGE

Poisoning by goose grease . 76
Pregnancy, fallibility of symptoms of . . . 234
——— to be detected by auscultation . . . 233
Puerperal fever, new remedy for 458

Quinine, cheap substitute for . 526

Rabies said, by Mr. Youatt, to be never spontaneous in the dog 149
Rheumatism from irritation of the medulla spinalis . 211
———, new practice in . ib.
———, colchicum in . 172
Rhubarb, different kinds distinguished . . 82

Skin, on the perspiratory vessels of 35
Small and cow pox, identity of, proved by curious experiments 525
Spinal irritation, on . 155
——— productive of hysteria, &c. . . 156
———, symptoms of 157
———, cases of 159, 163
——— producing hysteria 77
Spine, fracture of, without injury to the muscular power . 291
———, on distortions of . 155
Styptic, a new . . 265
Sudatory, Mr. Le Beaume's portable . . 529
Sweat, bloody, during hysteria 249
Symptoms, necessity of caution in the estimation of, in the last stages of some diseases . 137
Syphilis, cases of hereditary . 525
Syphilitic, curious cases of pseudo-, 454

PAGE

Tetanus, fatal case of traumatic; symptoms proceeding from the wound to the trunk . 312
———, on a species of intermittent 194
Tic douloureux from preternatural growth of bone . . 141
Toothach, remedy for . 171

University of London, annual examinations at . . 85
Uterine inflammation in puerperal women . . 317
———, post-mortem examination of thirty-four cases of . . . 318
———, treatment of 328
——— phlebitis, fatality of . 168
———, treatment of 169
Uterus, inflammation and softening of muscular tissue of, in puerperal women . 322
——— of veins and absorbents of, in puerperal women . . 323
———, recovery after laceration of . . . 127
———, attempt to account for the occurrence of inflammation of veins of, after delivery . 325

Vagina and uterus, recovery after laceration of, in labour . 128
Variola, chlorate of lime a preservative against . 253
Veins, obliteration of, causing œdema, &c. . . 109
———, inflammation of uterine, in puerperal women . 323
"Veterinarian," notice of the . 184

---

COLLECTANEA . . 76, 163, 247, 349, 435, 525
INTELLIGENCE . . 84, 179, 265, 355, 447, 527
METEOROLOGICAL REGISTER . 90, 186, 274, 362, 450, 538

### CRITICAL ANALYSES.

PAGE
Cholera, its Nature, Causes, and Treatment; with original Views, Physiological, Pathological, and Therapeutical, in relation to Fever; the Action of Poisons on the System, &c. By Charles Searle, Surgeon . 46
Treatise on the Excision of Diseased Joints. By James Syme, F.R.S. E. 60
An Essay on the Influence of Temperament in Modifying Dyspepsia. By Thomas Mayo, M.D. &c. . . . . 69
Essays and Orations, read and delivered at the Royal College of Physicians; to which is added, an Account of the Opening of the Tomb of King Charles I. By Sir Henry Halford, Bart., M.D., G.C.H. &c. . 130
On Canine Madness. By W. Youatt, V.S. and F.Z.S. . . 144
Observations on Distortions of the Spine; with a few Remarks on Deformities of the Legs. By Lionel J. Beale, Esq. . . . 155
Outlines of Physiology, &c. By W. P. Alison, M.D. F.R.S. E. &c. 227
Auscultation the only unequivocal Evidence of Pregnancy; with Cases. By J. C. Ferguson, A.M. M.B. . . . . 233
History of the Epidemic Cholera of Russia. By B. Hawkins, M.D. 242
Essay on Cholera Morbus. By J. Austin, Surgeon . . ib.
Pathological and Practical Researches on Uterine Inflammation in Puerperal Women. By Robert Lee, M.D., F.R.S., &c. . . 317
An Account of a Contagious Fever, which occurred amongst the Danish and American Prisoners of War at Chatham, in the Years 1813, 1814. By Sir Wm. Burnett, Knt., M.D., K.C.H., &c. . . . 331
First Principles of Medicine. By Archibald Billing, M.D. . 337
Principles of Lithotrity. By Baron Heurteloup . . 410
Medico-Chirurgical Notes and Illustrations. Part I. By R. Fletcher, Esq. 419
A Systematic Arrangement of British Plants, by Wm. Withering, M.D. Corrected and condensed by W. Macgillivray, A.M. . 429
A Manual of Medical Jurisprudence, compiled from the best Medical and Legal Works. By Michael Ryan, M.D. . . . . 492
The History of the Contagious Cholera. By James Kennedy, M.R.C.S. 504
A Treatise on the Diseases of the Heart and Great Vessels. By J. Hope, M.D. 513

### BIBLIOGRAPHICAL NOTICES.

The Study of Medicine, Surgery, and Anatomy; from the Creation of the World to the commencement of the 19th Century. By W. Hamilton, M.B. 89
The Veterinarian, published monthly . . . 184
Elements of Practical Midwifery. By Charles Waller, Esq. . 268
Advice to the Public for the Prevention and Cure of the Asiatic Cholera. From the German of Dr. J. R. Lichtenstadt . . 268
Recherches sur la Traitement du Cancer, par la Compression Méthodique, simple ou combinée, et sur l'Histoire générale de la même Maladie, &c. By J. C. A. Recamier, Médecin de l'Hôtel Dieu de Paris, &c. . 269
A Popular Treatise on the Teeth and Gums, and Diseases attendant on them, for the use of Families. By John Winckworth . . 272
The Art of Cupping. By G. F. Knox . . . . ib.
An Attempt to simplify the Treatment of Sexual Diseases. By James Thorn, Surgeon . . . . . . ib.
Observations and Experiments on a New Mode of Treating Fractures of the Leg and Fore Arm, especially Compound Fractures. By W. Beaumont, 359
Observations on Cholera, as it appeared at Port-Glasgow, during the Months of July and August, 1831; with Cases. By John Marshall, M.D. 431
A Treatise on Auscultation, illustrated by Cases and Dissections. By Robt. Spittal, House Surgeon, Royal Infirmary of Edinburgh . 433

PAGE
AUR. COR. CELSUS on Medicine, in eight Books, Latin and English. Translated from L. TARGA's Edition; the Words of the Text being arranged in the Order of Construction. Designed to facilitate the Progress of Medical Students. By ALEXANDER LEE, A.M. Surgeon . . 433
Operative Surgery. MAINGAULT's Illustrations of the different Amputations performed on the Human Body, represented by Plates designed after Nature; with Alterations and Practical Observations. By WM. SANDS COX, Lecturer on Anatomy and Surgery at Birmingham . . 434
A Practical Treatise on Injuries of the Head . . 522
Elements of Botany. Second Edition. By JOHN STEGGALL, M.D. &c. ib.
The Elements of Chemistry, familiarly explained. Part I.; numerous cuts 524

### NAMES OF AUTHORS,

*Of whose Works, Observations, &c. a more or less detailed Account is given in this Volume.*

ADAMS, FRANCIS, Esq. on the Opinions of the Ancients on Cholera . 363
ALISON, W. P., M.D. F.R.S.E. Outlines of Physiology . . 227
AUSTIN, JOHN, notice of his Essay on Cholera . . . 247

BARRY, Dr. DAVID, account of Russian Cholera . . 267
——————, on Russian Cholera . . 356. 448
——————, despatches from Russia relative to the Cholera 182
——————, on the Gibraltar Epidemic . . . 1. 96
BEALE, L. J., Esq. on Distortions of the Spine (analysis of) . 155
BEAUMONT, WM., Esq. notice of his Observations and Experiments on a New Mode of Treating Fractures . . 359, 447
BEECHEY, Captain, on the Habits of Mexican Domestic Bees . 82
BEER, Professor, on the Influence of Atmospheric Electricity on the Eyes 76
BILLING, ARCHIBALD, M.D. analysis of his First Principles of Medicine 337
BONAFOUS, M. on a New Styptic . . . . 265
BOW, Dr. on the External Use of Opium in Inflammatory Diseases . 23
BURN, R., Esq. on the External Use of Opium in Inflammatory Diseases 192
BURNETT, Sir W., knt. analysis of his Account of a Contagious Fever 331

CAILLARD, M. on Uterine Phlebitis . . . 168
CHAUFFORD, M. case of Bloody Sweat during Hysteria . . 249
CHERVIN, M. Experiment to determine whether Cholera may be imported by Merchandise . . . . . 248
CIVIALE, M. Report of Lithotritic Cases . . . 258
CLOUGH, Mr. on Chlorate of Lime in Gonorrhœa . . 170
COLLINS, Dr. R. cases of Recovery from Laceration of the Uterus and Vagina 127
COMBETTI, M. account of an Anatomical Phenomenon . . 247
CORBIN, Dr. Remarks tending to elucidate the History of Erysipelas 116
—————— on Œdema from Obliteration of Veins . . 109
COX, WM. SANDS, notice of his Translation of Maingault's Illustrations, &c. of different Amputations . . . . 434
CRAMPTON, J., M.D. case of Melanosis . . . . 224

DANCE, M. on a Species of Intermittent Tetanus . . 194
DARWALL, Dr. J. on Dependence of Hysteria upon Cerebral and Spinal Irritation . . . . . . . 77
DUFOUR, M. case of Death from Inanition . . . 436
DUPUYTREN, M. fatal case of Tetanus; Symptoms proceeding from the Wound towards the Trunk . . . . . 312

FERGUSON, J. C., A.M. M.B. analysis of his Essay, "Auscultation the only unequivocal Evidence of Pregnancy" . . 233
FLETCHER, R., Esq. review of his Medico-Chirurgical Notes and Illustrations, 419
—————— case of Peritonitis with Hernia . . 407

PAGE

FLETCHER, R., Esq. case of Strangulated Scrotal Hernia, produced by an Injury from the Patient's Watch . . . . 408
——— Strangulated Scrotal Hernia, &c. . 314
——— Encysted Hydrocele of the Cord, mistaken for a Rupture . . . . . . 315
FRISTO, Dr. cases of Poisoning by the Topical Use of Arsenic . 265

GEIGER, M. on Distinction of different Kinds of Rhubarb . 82
GERARDIN, M. on Ergot of Rye . . . . 258
GERHARD, M. case of Narcotism from Absorption . . 435
GIADOROU, Dr. on a cheap Substitute for Quinine . . 526
GRANT, NATHANIEL, Esq. cases of Indian Cholera . . 275
GREGORY, J. C., M.D., F.R.S. E. case of Peculiar Black Infiltration of the Lungs resembling Melanosis . . . 402
GUTHRIE, G. J., F.R.S. case of Gonorrhœal Ophthalmia . . 489
——— on Professional Education . . 444
——— cases of Femoral Aneurism opened by mistake 352
GUYON, M. case of Lacteal Infection . . . . 249

HAASE, Professor, on Hereditary Syphilis . . . 525
HALFORD, Sir HENRY, Bart., M.D. G.C.H. review of his Essays and Orations 130
HAKE, Dr. on the Perspiratory Vessels of the Skin . . 35
HAMILTON, WM., M.D. notice of his Study of Medicine, Surgery, and Anatomy, from the Creation of the World to the 19th Century 89
HAWKINS, BISSET, M.D. History of the Russian Cholera . . 242
HERZBERG, Dr. on Use of Chlorate of Lime in Purulent Ophthalmia 251
HEURTELOUP, Baron, review of his Principles of Lithotrity . 410
HOPE, J., M.D. review of his work on Diseases of the Heart . 513
HUFELAND, Professor, on the Use of Ointment of Mezereon to maintain a Discharge from Issues, &c. . . . . . 264

KENNEDY, JAMES, Esq. review of his work on Cholera . 504
KNOX, Mr. G. F. notice of his Art of Cupping . . . 272

LAIDLAW, JAMES, Esq. case of Fungus Hæmatodes of the Brain, with long-continued Constipation . . . . . 400
——— Surgical and Pathological Cases . . 451
——— case of Fracture of the Spine, &c. . 290
LARREY, Baron, answer to M. CIVIALE's Report of Lithotritic Cases 258
LAW, ROBERT, A.M. M.B. &c. on Hæmatemesis dependent upon Disease of the Liver . . . . . . 349
LE BEAUME, Mr. notice of his Portable Sudatory . . 529
LEE, ALEXANDER, A.M. Surgeon, notice of his Edition of CELSUS . 433
LEE, ROBERT, M.D. F.R.S., on Uterine Inflammation in Puerperal Women 317
LEE, EDWIN, Esq. on Italian Medical Institutions, &c. 301, 381, 461
LICHTENSTADT, M. case of Hydrothorax in a Child fifteen Months old 163
LICHTENSTADT, Dr. J. R. notice of his popular Essay on Cholera . 268
LONG, EDWIN, Esq. on the Treatment of Puerperal Fever . 458
LUIGI, SEMENTINI, Cavaliere, remarkable case of Coryza Phlegmatica 249

MACGILLIVRAY, W., A.M. review of his Edition of WITHERING's Systematic Arrangement of British Plants . . . 429
MACMICHAEL, W., M.D. notice of his Letter to Sir H. HALFORD, entitled "Is the Cholera a Contagious Disease?" . . 504
MALAGODI, Dr. Section of Sciatic Nerve for obstinate Neuralgia . 263
MARSHALL, J., M.D. notice of his Observations on Cholera, as it appeared at Port-Glasgow . . . 431
MAYO, HERBERT, F.R.S. case of Femoral Aneurism 39
——— Exostosis of the First Rib, with strong Pulsation of Subclavian Artery . . . 40
——— Lithotomy . . . 41
——— Disease of the Subcutaneous Bursa of the Patella . . . . . . ib.
——— Clinical Lecture on Hernia . . 485

PAGE

Mayo, Thomas, m.d. Essay on the Influence of Temperament in Modifying Dyspepsia . . . . . . 69
Menière, P., d.m.p. cases of Emphysema . . . 292
Mitchell, C. Esq. on Exposure to Mercurial Vapours . . 394
—————— on Pathology of Angina Pectoris . . 30
Mitchell, J. K., m.d. on a New Practice in Rheumatism . . 210

Nash, Mr. D. W. his Examination at the London University . 86
Neill, Hugh, Esq., m.r.c.s. Ed. on the Use of the Nitrate of Silver Ointment in Chronic Conjunctival Inflammation and in Gonorrhœa 259

O'Shaughnessy, Dr. on Poisoned Confectionary . . 178
—————— his Discovery of a Mode of distinguishing between the Meconate and Sulphocyanate of Iron . . 179

Pearson, E. S., Esq. Essay on the Principles of Medicine . 203
Perry, Dr. cases of Injuries of the Head . . . . 219
—————— immediate Amputation after Injuries . 222
—————— Aneurism . . . . 121
Prevost, M. on the Regeneration of Nervous Tissue . . 435

Recamier, Dr. J. C. A. notice of his Recherches sur la Traitement du Cancer par la Compression . . . . . 269
Remy, M. on Prevention of Smallpox and Measles by Chloride of Lime 253
Riecken, Dr. remarkable instance of Hemorrhagic Tendency in a Family 437
Rousseau, M. on the Cure of Fever by Holly-leaves . . 252
Russel, Dr. despatches from Russia relative to the Cholera . 182
—————— on Russian Cholera . . . . 356, 448
Russell, Mr. J. Description of his Fracture Box . . 91
Ryan, Dr. Remedy for Toothach . . . . 171
—————— review of his Manual of Medical Jurisprudence . 492

Sanson, M. his Practice in Uterine Phlebitis . . 170
Schlemm, Professor, Discovery of the Nerves of the Cornea . 247
Searle, Mr. Chas. on Cholera (analysis) . . . 46
—————— on the Cholera at Warsaw . . . 183
Siedler, Dr. cases of Poisoning by Goosegrease . . 76
Sonderland, Dr. on the Identity of Smallpox and Cowpox . 525
Spittal, R., Esq. notice of his Treatise on Auscultation . 433
Steggall, J., m.d. notice of his Elements of Botany . . 522
Stokes, Wm., m.d. case of Probable Dislocation of the Heart from external Violence . . . . . . 440
Swift, J. K., m.d. case of Fracture of the Cranium, with Loss of Brain and Hernia Cerebri: Recovery . . . . 261
Syme, Jas., f.r.s. e. on Excision of Diseased Joints . . 60

Taylor, J. R., Esq. on the Nature and Cause of tbe Buffy Coat of the Blood, 187
Thomas, W. J., Esq. on Consecutive Diseases of Dyspepsia . 478
Thomson, Dr. A. T. Questions proposed by him at the London University 86
Thorn, Mr. Jas., Surgeon, notice of his Attempt to Simplify the Treatment of Sexual Diseases . . . . 272

Tweedie, Dr. A. on Colchicum in Rheumatism . . 172

Waller, C. Esq. notice of the second Edition of his Elements of Practical Midwifery . . . . . . 268
—————— Report of Midwifery Cases . 109
Wark, Mr. David, cases of Spinal Irritation . . . 163
Whatton, W. R., Esq. on Pertussis . . 254
Winckworth, Mr. John, notice of his Treatise on the Teeth . 272
Youatt, W., v.s. and f.z.s. review of his Essay on Canine Madness 144

C. Adlard, Printer, Bartholomew Close.

